# Cocaine promotes oxidative stress and microglial-macrophage activation in rat cerebellum

**DOI:** 10.3389/fncel.2015.00279

**Published:** 2015-07-28

**Authors:** Rosa López-Pedrajas, Dolores T. Ramírez-Lamelas, Borja Muriach, María V. Sánchez-Villarejo, Inmaculada Almansa, Lorena Vidal-Gil, Francisco J. Romero, Jorge M. Barcia, María Muriach

**Affiliations:** ^1^Instituto de Ciencias Biomédicas, Departamento de Ciencias Biomédicas, Universidad CEU Cardenal HerreraMoncada, Valencia, Spain; ^2^Structure and Function of the Human Body, Facultad de Medicina y Odontología, Universidad Católica de Valencia ‘San Vicente Mártir,’ ValenciaSpain; ^3^UP Medicina, Facultad de Ciencias de la Salud, Universitat Jaume I, CastellónSpain

**Keywords:** cerebellum, cocaine, nuclear factor kappa B, oxidative stress, mononuclear phagocyte

## Abstract

Different mechanisms have been suggested for cocaine neurotoxicity, including oxidative stress alterations. Nuclear factor kappa B (NF-κB), considered a sensor of oxidative stress and inflammation, is involved in drug toxicity and addiction. NF-κB is a key mediator for immune responses that induces microglial/macrophage activation under inflammatory processes and neuronal injury/degeneration. Although cerebellum is commonly associated to motor control, muscular tone, and balance. Its relation with addiction is getting relevance, being associated to compulsive and perseverative behaviors. Some reports indicate that cerebellar microglial activation induced by cannabis or ethanol, promote cerebellar alterations and these alterations could be associated to addictive-related behaviors. After considering the effects of some drugs on cerebellum, the aim of the present work analyzes pro-inflammatory changes after cocaine exposure. Rats received daily 15 mg/kg cocaine i.p., for 18 days. Reduced and oxidized forms of glutathione (GSH) and oxidized glutathione (GSSG), glutathione peroxidase (GPx) activity and glutamate were determined in cerebellar homogenates. NF-κB activity, CD68, and GFAP expression were determined. Cerebellar GPx activity and GSH/GSSG ratio are significantly decreased after cocaine exposure. A significant increase of glutamate concentration is also observed. Interestingly, increased NF-κB activity is also accompanied by an increased expression of the lysosomal mononuclear phagocytic marker ED1 without GFAP alterations. Current trends in addiction biology are focusing on the role of cerebellum on addictive behaviors. Cocaine-induced cerebellar changes described herein fit with previosus data showing cerebellar alterations on addict subjects and support the proposed role of cerebelum in addiction.

## Introduction

Cocaine effects have been widely studied in neural areas traditionally related with drug addiction e.g., frontal cortex, NAc, and VTA among others. Cerebellum has been typically involved in functions related to motor control, from balance to fine motor tuning. However, some studies indicate that cerebellum could be involved in higher cognitive processes (Ramnani, 2006; Izawa et al., 2012). Even more, there are evidences supporting the idea that cerebellum is also involved with cocaine addiction (Anderson et al., 2006; Sim et al., 2007; Vazquez-Sanroman et al., 2015).

Much of the interest in the role of the cerebellum in cognition is based on the presence of anatomical pathways connecting the cerebellum and dorsolateral prefrontal cortex (Middleton and Strick, 1994). In addition, cerebellar lesions lead to behavioral changes characterized by executive function impairments such as motor planning, verbal fluency, and changes in personality among others. Moreover, it has been suggested that some of these changes implicate altered functional connections between cerebellum and prefrontal, posterior parietal, temporal, and limbic cortices (Jiménez-Rivera et al., 2000).

Recent studies indicate that cocaine exposure promotes molecular and structural cerebellar alterations (Palomino et al., 2014; Moreno-López et al., 2015) and fitting with this, some neuroimaging studies show how cocaine alters cerebellar functions (Anderson et al., 2006; Sim et al., 2007).

There is a growing body of evidence implicating oxidative stress in the pathogenesis and development of addiction to cocaine and other substances (Uys et al., 2011; Sordi et al., 2014). Despite oxygen is essential for aerobic life, and particularly central nervous system exhibits the highest oxygen consumption rate (20% from total O_2_), excessive amounts of ROS derived from cell activity can result deleterious for cells. As other addictive substances, cocaine promotes oxidative stress in specific areas closely implicated in the circuitry of addiction such as NAc, frontal cortex, and hippocampus (Dietrich et al., 2005; Muriach et al., 2010; Jang et al., 2014). Surprisingly there is no literature about how cocaine modulates oxidative stress in cerebellum.

As occurs during inflammatory processes, ROS recruit inflammatory responses and microglia/macrophage, referred as MP, represents one of the most relevant actors during this process (Aguzzi et al., 2013). During pathological processes, MP releases cytokines, trophic factors, and even ROS (Krasnova and Cadet, 2009), and as reviewed by McNally et al. (2008) these pro-inflammatory factors, such as ROS and cytokines, impair astrocytic glutamate reuptake, resulting neurotoxic for cells. Stressing this hypothesis, cocaine exposure leads to up-regulation of pro-inflammatory mediators such as cytokines and chemokines, or astroglia/microglia activation (Renthal et al., 2009; Piechota et al., 2010; Blanco-Calvo et al., 2014).

In addition, one of the most relevant roles of MP is that related to phagocytosis of pathogens, degenerating cells and debris (Schafer et al., 2013). Apart from this defense rol, resident microglia is also involved in other physiological processes such as neuronal activity modulation, synaptic regulation, learning, and memory (Wake et al., 2009; Tremblay et al., 2011; Pascual et al., 2012; Blank and Prinz, 2013). Microglia has been also implicated in the development of cocaine addiction (Del Olmo et al., 2007; Porter et al., 2011). Despite of there are controversial results about MP activation during cocaine exposure (Little et al., 2009; Narendran et al., 2014), psychostimulant drugs activate specific components of the response, following both acute and chronic psychostimulant exposure (Yamamoto et al., 2010). Several researchers state that methamphetamine induces microglial activation in the brain (Thomas et al., 2004). Moreover, reactive microgliosis (estimated as microglia/macrophage activation) has been detected in several brain regions of methamphetamine addicts even after years of abstinence (Sekine et al., 2008). On this line, some reports indicate that cerebellar MP activation induced by cannabis or ethanol could be associated to cerebellar impairments (Cutando et al., 2013; Drew et al., 2015). These alterations fit with the proposed implication of cerebellum in the development of addictive-related behaviors.

Nuclear factor kappa B is a central mediator of human immune response (Pahl, 1999), and acts as sensor of oxidative stress situations (Schreck et al., 1992). In addition, NF-κB-DNA binding activity and transcription are regulated by various forms of synaptic activity (Albensi and Mattson, 2000; Freudenthal et al., 2004). In fact, O’Riordan et al. (2006) provide evidence suggesting that hippocampal NF-κB is activated by metabotropic glutamate receptors and Willard and Koochekpour (2013) proposed that NF-κB-mediated glutamate signaling plays a role in many neuronal processes where synaptic remodeling and plasticity are critical, e.g., learning and memory. Moreover, activation of group II mGluRs by glutamate promotes TNF release and neurotoxicity by activating NF-κB signaling (Lee, 2013).

Nuclear factor kappa B has been proposed as target of drugs such as ethanol or cocaine. Thus, increased NF-κB activity in the NAc after cocaine exposure has been demonstrated (Ang et al., 2001; Russo et al., 2009). Furthermore, ethanol exposure induces NF-κB activity in the mouse brain, which in turn, induces the transcription of pro-inflammatory immune genes, increasing expression of cytokines, proteases, and oxidases (Qin et al., 2008; Mayfield et al., 2013).

So, after considering that cocaine increases oxidative stress in several brain areas and the relevant role of NF-κB in oxidative stress, inflammation, and addiction. This report is addressed to investigate the oxidative cerebellar-related misbalance induced by cocaine giving more support to the emerging hypothesis that implicates cerebellum in addiction.

## Materials and Methods

### Experimental Design

Male Wistar rats weighing 300 g (Charles River Laboratories SA, Barcelona, Spain) were used for the experiment (*n* = 20). Rats were individually caged and maintained in a 12 h light/dark cycle with controlled temperature (20–25°C) and relative humidity (60%). Animals had access to food and water *ad libitum*. All the experimental procedures were carried out according to the European Union regulation (order 86/608/CEE) and approved by the committee on animal care of the UCH-CEU with reference number 11/022.

The experiment lasted 18 days. In this period, the animals were separated into two groups, control and cocaine. Cocaine was daily administered by intraperitoneal injection at a dose of 15 mg/Kg in saline, and control animals received same saline volume (0.9%) in the intraperitoneal cavity.

Rats were sacrificed on the last day by cervical dislocation (previously anesthetized with pentobarbital). Brains were removed and cerebellum was dissected and divided into two parts. One part was used to measure oxidative stress markers and glutamate concentration. Samples used to analyze the oxidative stress were homogenized in 0.1 M PB (HK_2_O_4_P 0.1 M; H_2_KO_4_P 0.1 M) pH 7.0 at 4°C.

Homogenates were centrifuged at 6,000 rpm 2 min and the supernatant was stored at -20°C until used for protein determination and glutathione peroxidase (GPx) enzyme activity. In the case of GSH, GSSG, and glutamate, immediately after centrifugation, the supernatant was acidified with 20% of perchloric acid (Panreac, Spain) and stored at –20°C until the determination of these parameters.

Protein content was measured by means of the Lowry method (Lowry et al., 1951) to allow expression of the biochemical results taken into account the protein content of each sample.

Samples for western blot analysis and NF-κB activity. Nuclear fraction was separated from cytoplasmic fraction using the following protocol. Cerebellar tissue was homogenized on working solution A (Hepes 10 mM, KCl 5 mM, EDTA 0.1 mM, EGTA 0.1 mM, Ditiotreitol 100 mM, IGEPAL 0.05%, Complete 1x, NaF 10 mM, Na_3_V0_4_ 200 mM). The samples were centrifuged at 850*g* for 10 min at 4°C. The supernatant was separated from the pellet and stored. 400 μl of working solution A were mixed, incubated 15 min at 4°C and centrifuged at 10,000*g* for 30 s at 4°C. The supernatant was collected and stored (cytoplasmic fraction). Hundred and fifty microliter of the working solution C were added to the remaining pellet (Hepes 20 mM, NaCl 0.4 M, EDTA 1 mM, EGTA 1 mM, Ditiotreitol 100 mM, Complete 1x, NaF 10 mM, Na_3_V0_4_ 200 mM) and incubated again 15 min at 4°C. The sample was centrifuged at 10,000*g* for 5 min at 4°C. The collected supernatant was the nuclear fraction. Both cytoplasmic (used for western blot analysis) and nuclear fractions (used to measure NF-κB activity) were stored at -20 for later use.

Animals assigned for immunohistochemical procedures were perfused with saline followed by 4% paraformaldehyde solution in 0.1 M phosphate buffer (PB), pH 7.4. Brains were post-fixed in paraformaldehyde solution for 24 h and then placed in a 30% sucrose solution for 24 h. Thirty micrometer thick sections were obtained using cryostat.

### Oxidative Stress

Reduced GSH, GSSG, and glutamate concentrations were quantified following the method of Reed et al. (1980), based in the reaction of iodoacetic acid with the thiol groups followed by a chromophore derivatization of the amino groups with Sanger reactant (1-fluoro-2,4-dinitrobencene), giving rise to derivates which are quickly separated by means of high-performance liquid chromatography (HPLC).

Glutathione peroxidase activity, which catalyzes the oxidation by H_2_O_2_ of GSH to its disulfide (GSSG), was assayed spectrophotometrically as reported by Lawrence et al. (1978) toward hydrogen peroxide, by monitoring the oxidation of NADPH at 340 nm. The reaction mixture consisted of 240 mU/mL of GSH disulfide reductase, 1 mM GSH, 0.15 mM NADPH in 0.1 M potassium phosphate buffer, pH 7.0, containing 1 mM EDTA and 1 mM sodium azide; a 50 μL sample was added to this mixture and allowed to equilibrate at 37°C for 3 min. Reaction was started by the addition of hydrogen peroxide to adjust the final volume of the assay mixture to 1 mL.

### Western Blot Analysis

Samples were resolved on 10% SDS polyacrylamide gels and transferred to nitrocellulose membrane. Membranes were blocked in 5% skim milk in T-TBS buffer and 0.1% Tween 20, for 1 h and were incubated thereafter with the primary antibody overnight at 4°C. Primary antibodies used were ED1 (a lysosomal protein which is overexpressed during inflammatory challenge, and is used as a marker to confirm microglial activation), peroxidase β-Actin (Sigma–Aldrich, Alcobendas, Spain) caspase 3 (a pro-apoptotic protein; Cell Signaling, Barcelona, Spain) and GFAP one of the major intermediate filament proteins of mature astrocytes (Dako, Denmark). Peroxidase-coupled secondary antibodies were used for primary antibody detection by incubating membranes 1 h at room temperature. (anti-mouse Thermo Fisher Scientific, Madrid, Spain; anti-rabbit, Santa Cruz, California, EEUU) Finally, the signal was detected with ECL developing kit (Amersham Biosciences, UK). Blots were quantified by densitometry using Quantity One software and the results were represented in density units.

Ba/F3 cells (murine interleukin-3 dependent pro-B cell line) were used as positive control for caspase-3 activation.

### CD68 (ED1) Immunohistochemistry

Cerebellar CD68 expression was examined by immunohistochemistry. Sections were rinsed with 0.01M PBS, pH 7.0 and blocked with 30% H_2_O_2_ for 20 min followed by incubation overnight with a primary rabbit anti-CD68 (ED1) (Abcam, Cambridge, UK; dilution 1:500 in PBS with 0.3% Triton X-100 and 5% normal goat serum). Sections were rinsed in PBS and incubated at room temperature shaking for 1 h in 0.4% biotinylated anti-rabbit IgG. Finally, sections were rinsed and re-incubated with avidin-biotin complex and reaction was developed with DAB.

Images were captured with a CCD camera (Coolsnap FX Color; Roper Scientific).

### Nuclear Factor Kappa B Activity

To determine NF-κB activity in the nuclear fraction, an ELISA-based kit to detect and quantify transcription factor activation was used, TransAM NF-κB (Active Motif, Rixensart, Belgium). Results were represented as arbitrary units.

### Terminal Deoxynucleotidyl Transferase Biotin-dUTP Nick end Labeling

Transferase biotin-dUTP nick end labelling (TUNEL) assay was performed by an *in situ* cell death detection kit (Roche Diagnostics, Mannheim, Germany), according to the manufacturer’s instructions. DNAase reaction was used for positive control labeling.

### Statistical Analysis

Results are presented as mean values ± SE. Statistical significance was assessed by Students *t-*test. The level of significance was set at *p* < 0.05.

## Results

### Antioxidant Defenses are Decreased in the Cerebellum After 18 days of Cocaine Administration in Rats

**FIGURE 1:**
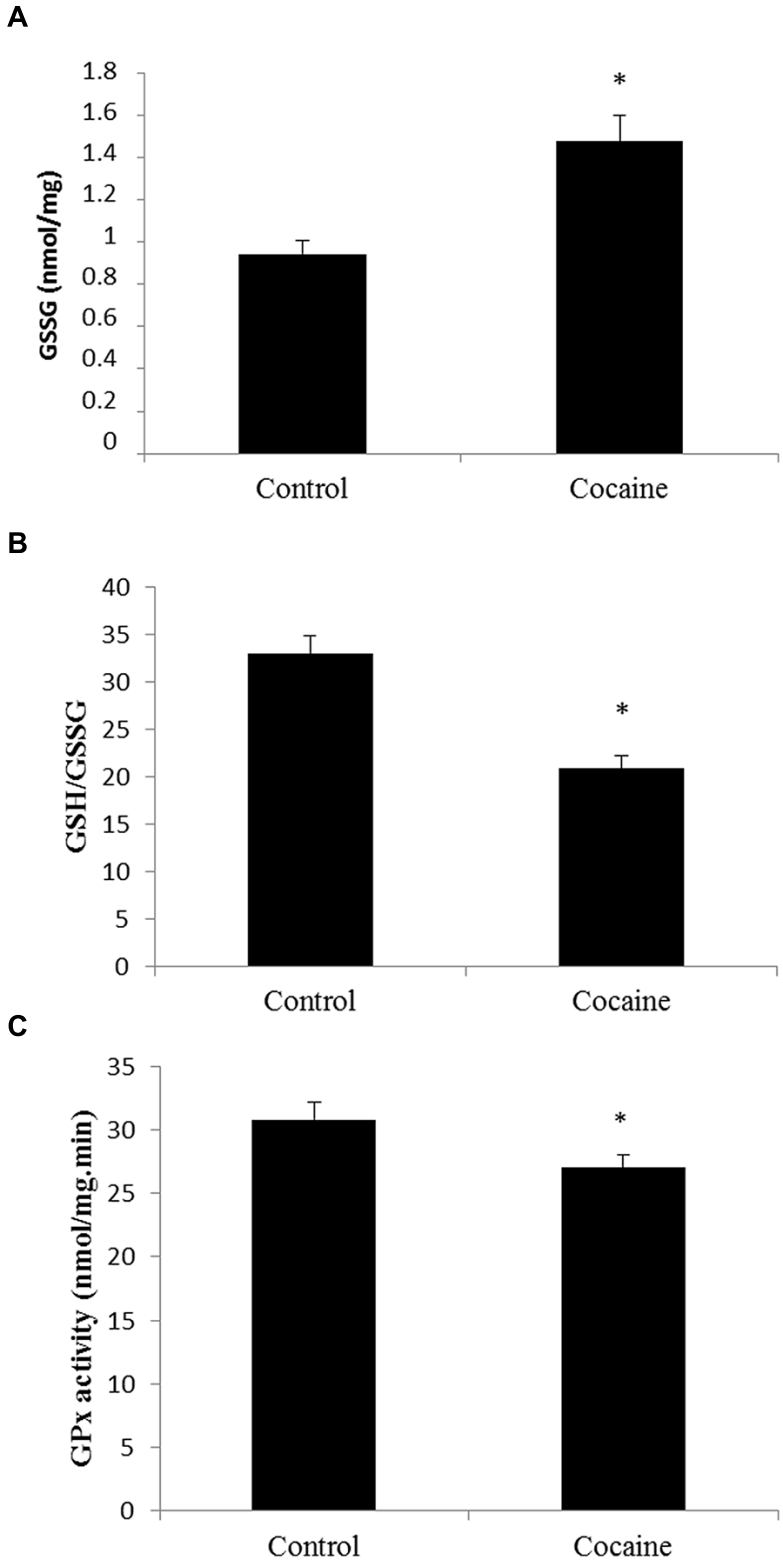
**Effect of cocaine administration on cerebellar antioxidant defenses. (A)** GSSG concentration (nmol/mg prot) *p < 0.05 vs. control group. **(B)** GSH/GSSG ratio *p < 0.05 vs. control group. **(C)** GPx activity (nmol/mg.min) *p < 0.05 vs. control group.

Cerebellar homogenates were processed for HPLC determination. Cerebellar GSH levels remained unaltered (data not shown) whereas GSSG levels presented a statistically significant increase compare to control GSSG levels (**Figure 1A**). Interestingly, the GSH/GSSG ratio was statistically significant lower in cerebella from cocaine treated animals compared to control animals (**Figure 1B**). GPx is the enzyme that converts GSH to GSSG by reducing hydrogen peroxide (H_2_O_2_) to water. Cocaine treated group presented statistically significant lower cerebellar levels of GPx than control groups (**Figure 1C**).

### Astrocytic and Microglial/Macrophage Response

**FIGURE 2:**
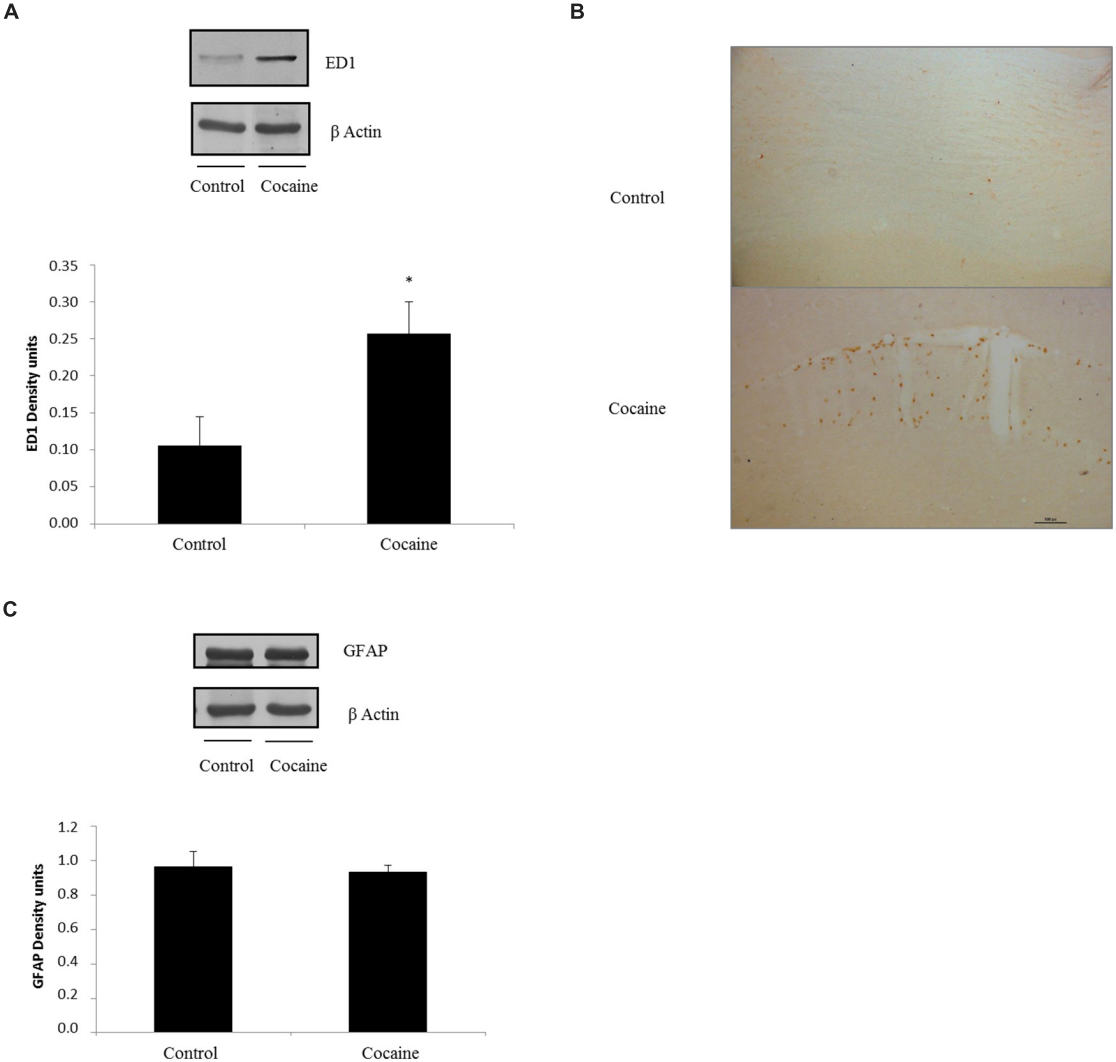
**ED1 (68 kDa) and β-Actin (42 kDa) western blot dots in rat cerebellum**. Representation of ED1 (density units) in cerebellum showing a significant ED1 increase in cerebellar samples of cocaine-treated rats (*p < 0.05 vs. control group; **A**). ED1 immunohistochemistry from control and cocaine-treated cerebellar samples. Little or null ED1 positive cells can be observed in control samples, whereas noticeable ED1 positive cells can be found around cerebellar vessels **(B)**. GFAP (50 kDa) and β-Actin (42 kDa) western blot dots in control and cocaine-treated cerebellar samples, with GFAP representation (as density units). No differences can be set between control and cocaine-treated rats suggesting a lack of astrocytic response after cocaine exposure **(C)**.

ED1 antigen or CD68 is a lysosomal protein expressed during inflammatory processes by both microglia and macrophages. Cerebellar CD68 expression was determined by western blot. As shown in **Figure 2A**, ED1 expression was statistically significant increased in cocaine treated rats when compared to control group.

Fitting with this result, ED1-IHC technique shows almost unappreciable rounded ED1 positive cells sparse and randomly located in the cerebellar cortex of control animals. However, cerebella from cocaine treated animals present more and rounded ED1 positive cells with evident perivascular location (**Figure 2B**).

In order to investigate the possibility of a pro-inflammatory response, glial fibrilary acidic protein (GFAP) was analyzed by western blot. This astrocytic protein is over expressed under inflammatory-related responses. Surprisingly no GFAP differences could be set between groups (**Figure 2C**).

### Glutamate Concentration

**FIGURE 3:**
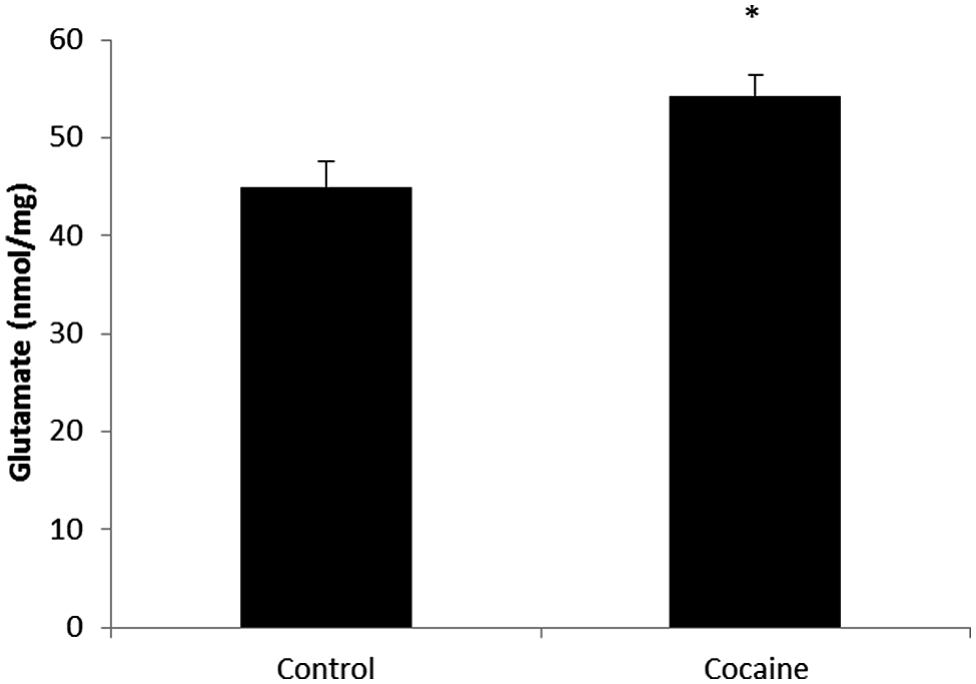
**Microglial-macrophage activation is closely related to extracellular glutamate over-drive.** HPLC cerebellar glutamate levels (nmol/mg of total protein) in control and cocaine-treated rats. Total cerebellar glutamate levels are increased after cocaine administration. (*p < 0.05 vs. control group).

Total glutamate (extra-cellular + intra-cellular) was measured by HPLC from cerebellar homogenates. As shown in **Figure 3**, 18 days of cocaine administration induced a statistically significant increase on cerebellar glutamate concentration compared to control rats.

### Nuclear Factor Kappa B Activity

Cytoplasmic IκB factor inhibits NF-κB. NF-κB inducing stimuli activate the IκB kinase complex that phosphorylates IκB. IκB degradation exposes the NF-κB DNA-binding domain allowing its nuclear translocation regulating NF-κB target genes.

**FIGURE 4:**
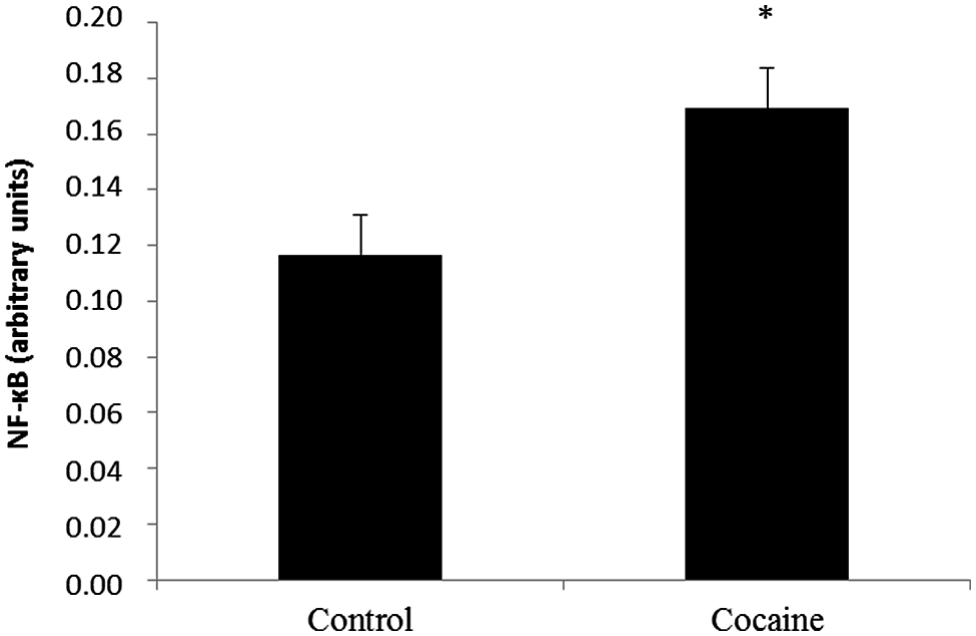
**Nuclear factor kappa B is a redox-sensitive nuclear factor involved in the control of immune-inflammatory responses.** Cerebellar NF-κB activity assay (arbitrary units) from control and cocaine- treated animals (18 days) showing a significant increase of NF-κB activity in cocaine-treated rats (*p < 0.05 vs. control group).

NF-κB activity from the nuclear fraction was statistically significant increased in cerebella of cocaine treated rats when compared to control rats (**Figure 4**).

### Apoptotic Cell Death: Caspase 3 Expression and TUNEL

**FIGURE 5:**
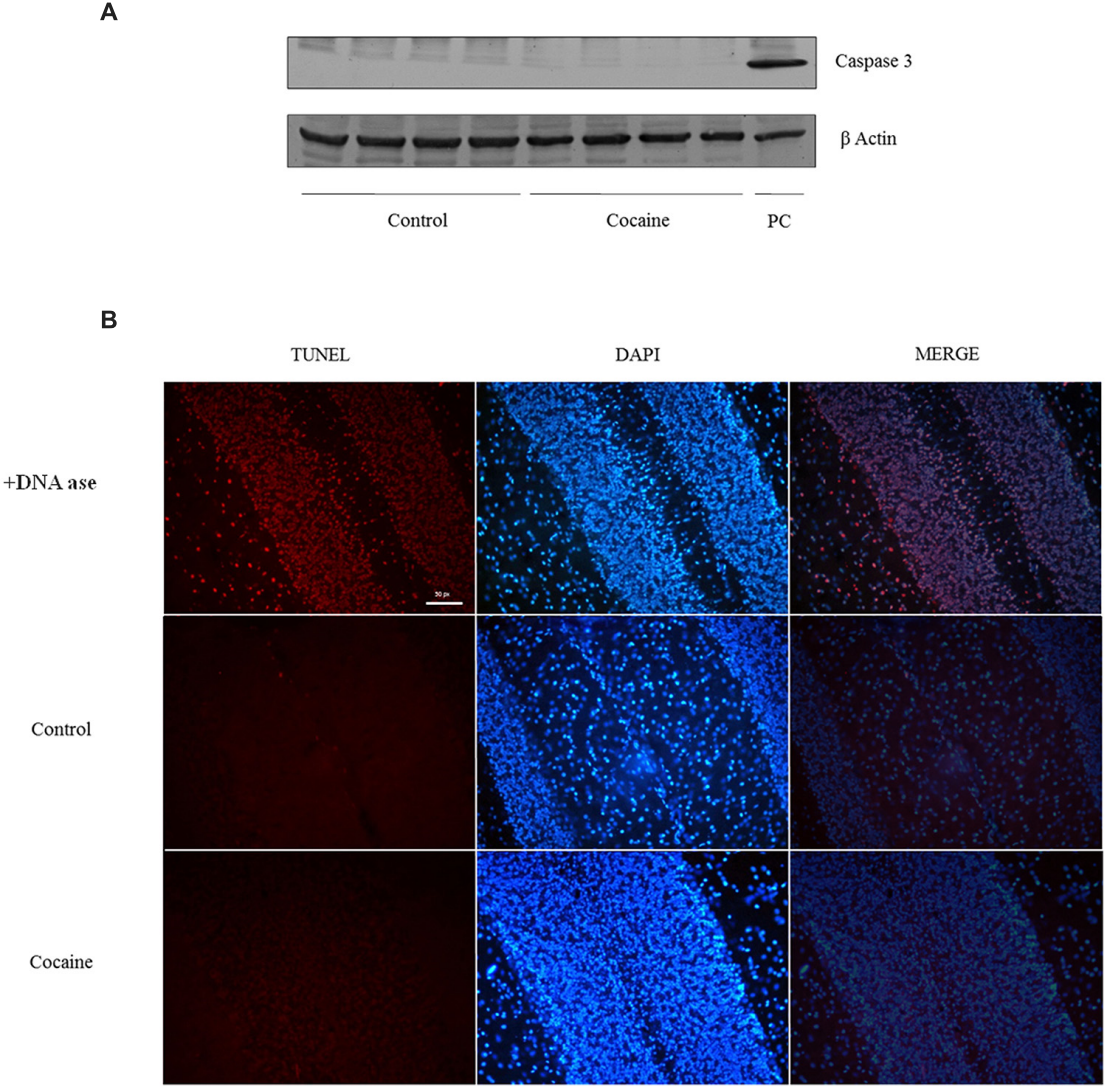
**Caspase-3 (17/19 kDa) and β-Actin (42 kDa) western blot analysis in cerebellum **(A**; Ba/F3 cells were used as positive control of caspase 3 activation).** TUNEL assay on cerebellum: +DNA ase reaction (used as positive control of TUNEL) exhibits profuse TUNEL-positive cell labeling (red dye). Minimal or null TUNEL-positive labeled cells could be demonstrated in control or cocaine-treated cerebellar samples **(B)**.

No signal of pro-apoptotic protein caspase-3 was observed in cerebellum after 18 days of cocaine exposure (**Figure 5A**). However, because caspase-3 is activated after its cleavage, TUNEL determination was conducted in cerebellar samples in order to find apoptotic cell death. As observed in TUNEL positive control sample (DNAase reaction) profuse TUNEL positive labeled cells can be found. Little or null TNEL positive labeling could be demonstrated in control or cocaine treated groups (**Figure 5B**).

## Discussion

### Mononuclear Phagocyte Activation and Glutamate Concentration

Mononuclear phagocyte activation has been related with psychiatric diseases (Holstege et al., 2008; Chen et al., 2010). Addiction is included as a mental disorder and therefore several studies are currently focusing on the structural and molecular CNS alterations during drug consumption and addiction. In this sense, large evidence indicates that different drugs from ethanol to psychostimulants (e.g., cocaine) promote microglial activation (Thomas et al., 2004; Little et al., 2009; Yamamoto et al., 2010; Raineri et al., 2012; Cutando et al., 2013; Drew et al., 2015). It is well documented that cocaine exposure affects microglia (Hayashi and Su, 2003), up-regulating pro-inflammatory mediators such as cytokines with astroglia/microglia activation (Renthal et al., 2009; Piechota et al., 2010; Blanco-Calvo et al., 2014).

Cocaine challenge resulted in a marked increase of cerebellar ED1. Fitting with this, ED1 is overexpressed during inflammatory challenge, and it is used as marker to confirm MP activation (Graeber et al., 1990). Furthermore, ED1 seems to correlate with the capacity of postnatal microglia to engulf synapses (Schafer et al., 2013). After consider the typical phagocytic role of microglia during inflammation-related processes and the role of microglia in remodeling neural contacts during learning and memory processes (Felger and Miller, 2012; Blank and Prinz, 2013), it seems plausible that cocaine-induced cerebellar MP activation might be related to these aforementioned cerebellar changes after cocaine exposure.

Fitting with the increased cerebellar glutamate levels found, cerebellar glutamatergic activation has been also described after cocaine exposure (McFarland et al., 2003; Palomino et al., 2014). Although synaptic glutamate results of high interest for addiction and learning-memory processes, the present report focus attention on extra-synaptic glutamate, in view of its relevance on inflammation and its implication with mental disorders (Müller and Schwarz, 2007). In this sense, MP activation is closely related to extracellular glutamate over-drive leading to neurotoxicity and neural remodeling. So, the finding of increased cerebellar glutamate levels fits with CD68 over expression and lends support to previous reports on cocaine-related cerebellar alterations in overt behavior and cognition (Jiménez-Rivera et al., 2000). One relevant observation is that related to cerebellar function after cocaine exposure. As previously reported, no differences could be found on targeting-directionality in the Morris water maze test after the same cocaine challenge (Muriach et al., 2010). So there are not evidences of cocaine-related cerebellar alterations after this experimental paradigm in terms of motor-related functions.

### Antioxidant Defenses and Apoptotic Markers

Some reports indicate redox status misbalance after cocaine treatment in several brain areas (Dietrich et al., 2005; Muriach et al., 2010; Uys et al., 2011). Interestingly and as novelty, this is the first report showing cerebellar oxidative alterations after cocaine challenge. It is well known that oxidative stress causes cellular damage and eventually cell death (Calabrese et al., 2007). Because caspase-3 levels were undetectable after cocaine challenge and caspase-3 is active after its cleavage, TUNEL assay was developed. The lack of TUNEL labeling indicates a lack of apoptotic cell death, suggesting that apoptosis is not promoted after cocaine exposure in cerebellum. Fitting with this, other reports show the absence of apoptosis after cocaine exposure in brain (Dominguez-Escriba et al., 2006; Muriach et al., 2010). Controversially, some reports indicate that cocaine exposure induces apoptosis in different tissues (Cerretani et al., 2012; Blanco-Calvo et al., 2014). The discussion about what dose or duration can promote cell death (apoptotic or not) is so far from the goal of this work and probably the aforementioned differences could be explained depending on the tissue, cocaine concentration/duration, animal model, etc. In fact, diverse published data are conducted with different cocaine doses and duration. For this work, cocaine dose and duration was chosen from previous published works (Ishikawa et al., 2009; Schroeder et al., 2009; Muriach et al., 2010).

Dopamine auto-oxidation (Numa et al., 2008) has been typically accepted as cocaine-induced ROS source. However, dopamine is not the main neurotransmitter in cerebellum, particularly present in vermis (Melchitzky and Lewis, 2000). Therefore, the observed antioxidant defense decrease could be due to other origins. Cocaine-induced vasoconstriction may decreases cerebellar blood flow, leading to hypoxia increasing ROS (Kaufman et al., 1998; Gottschalk and Kosten, 2002; Pae et al., 2005). Additionally, it has been described that cocaine increases brain temperature, which is a reliable indicator of metabolic neural activation (Kiyatkin and Brown, 2004) and thus, cocaine-enhanced metabolism can increase ROS (Brookes et al., 2004). Finally and fitting with the results shown herein, the decrease of antioxidant defenses could be due to MP activation, since activated microglia and macrophages can produce ROS after LPS stimulus (Marín-Teva et al., 2004; Di Penta et al., 2013). In conclusion, despite there are multiple ways by which cocaine potentially promotes oxidative stress. The findings shown herein indicate that cocaine-induced cerebellar MP activation is accompanied by antioxidant defense decay, suggesting an unusual pro-inflammatory response since GFAP is unaltered. Future studies must be addressed to resolve this issue.

### NF-κB is Activated After Cocaine Exposure

Nuclear factor kappa B is a redox-sensitive nuclear factor involved in the control of immune-inflammatory responses, developmental processes, and apoptosis. NF-κB is a key regulator of inflammation and secondary injury processes. In fact, several members of the NF-κB family are considered essential regulators of cellular activities associated with inflammation/chemokine production (Ghosh and Hayden, 2008). Stimuli such as cytokines, free radicals, ultraviolet irradiation, bacterial or viral antigens and glutamate increase NF-κB-DNA binding promoting chemokine-cytokine gene transcription (Schreck et al., 1992; Meffert and Baltimore, 2005). Although NF-κB is expressed in many cells, NF-κB is transcriptionally active primarily in glia (Mao et al., 2009). Astrocytes, monocytes, and microglia express high levels of NF-κB under pathological situations, this transcription factor is the key one involved in induction of innate immune genes in microglia and other monocyte-like cells (Mattson and Camandola, 2001; Crews et al., 2011). The lack of GFAP over expression may suggest that astrocytes are not directly involved in this NF-κB activation.

Nuclear factor kappa B activation seems to mediate some processes in cocaine addiction, particularly in the NAc, hippocampus, or frontal cortex (Ang et al., 2001; Russo et al., 2009), but nothing is known regarding other brain areas, such as the cerebellum. Cerebellar cocaine-induced NF-κB activation is accompanied by a significant decrease of the antioxidant defense and by increased microglial/macrophage ED1 expression. In line with this, increased p65 NF-κB activity accompanied by ROS production and cytokine expression has been also demonstrated in cocaine-treated microglia (Yao et al., 2010). Moreover, ROS promotes microglial NF-κB activation (Block et al., 2007).

### Relationship between NF-κB, Glutamate Concentration and Antioxidant Defenses

It is well known that NMDA receptors activate NF-κB (Lipsky et al., 2001; Munhoz et al., 2008). Therefore, the increase observed in NF-κB activity, could be due to the enhanced glutamate concentration observed after cocaine administration. Moreover, Barger et al. (2007) reported that the glutamate release from activated microglia is an indirect consequence of GSH depletion. Thus, it seems that if cocaine increases cerebellar glutamate concentration, it could be associated to GSH depletion. On the other hand, the activation of NMDA and AMPA receptor subtypes causes the mobilization of free cytosolic calcium, and the excess of intracellular calcium results in ROS generation (Carriedo et al., 1998).

Cocaine promotes oxidative cerebellar misbalance with increased ED1 expression, as estimation of mononuclear phagocytic activity. In addition, NF-κB activation and increased glutamate levels strongly suggest a pro-inflammatory process underlying mechanism after cocaine exposure. Future studies would be addressed to investigate the role and meaning of this cocaine-induced cerebellar ED1 over expression and whether these molecular and cellular modifications may lead to perpetuate neural circuitries involved in addiction.

## Author Contributions

MM, JB, and FR conceived and designed the study. RL-P, DT, and MS-V performed the experiments. IA, MS-V, and DR-L were responsible for the biochemical analysis. BM, RL-P, and DR-L performed the western blot analysis. JB, IA, and BM were responsible for the statistical analyses. LV-G and RL-P performed IHC procedures, RL-P and DR-L designed the figures. RL-P, JB, MM, and FR wrote the manuscript.

## Conflict of Interest Statement

The authors declare that the research was conducted in the absence of any commercial or financial relationships that could be construed as a potential conflict of interest.

## Abbreviations

GFAP: glial fibrillary acidic protein
GSH: glutathione
GSSG: oxidized glutathione
IκB: inhibitory kappa B
MP: mononuclear phagocyte
NAc: nucleus accumbens
NF-κB: nuclear factor kappa B
ROS: reactive oxygen species
VTA: ventral tegmental area.
